# A modular and adaptable analysis pipeline to compare slow cerebral rhythms across heterogeneous datasets

**DOI:** 10.1016/j.crmeth.2023.100681

**Published:** 2024-01-05

**Authors:** Robin Gutzen, Giulia De Bonis, Chiara De Luca, Elena Pastorelli, Cristiano Capone, Anna Letizia Allegra Mascaro, Francesco Resta, Arnau Manasanch, Francesco Saverio Pavone, Maria V. Sanchez-Vives, Maurizio Mattia, Sonja Grün, Pier Stanislao Paolucci, Michael Denker

**Affiliations:** 1 Institute of Neuroscience and Medicine (INM-6) and Institute for Advanced Simulation (IAS-6) and JARA-Institute Brain Structure-Function Relationships (INM-10), Jülich Research Centre, Jülich, Germany; 2 Theoretical Systems Neurobiology, RWTH Aachen University, Aachen, Germany; 3 Istituto Nazionale di Fisica Nucleare (INFN), Sezione di Roma, Rome, Italy; 4 Institute of Neuroinformatics, University of Zürich and ETH Zürich, Zürich, Switzerland; 5 European Laboratory for Non-linear Spectroscopy (LENS), University of Florence, Florence, Italy; 6 Neuroscience Institute, National Research Council, Pisa, Italy; 7 Department of Physics and Astronomy, University of Florence, Florence, Italy; 8 Institut d’Investigacions Biomèdiques August Pi i Sunyer (IDIBAPS), Barcelona, Spain; 9 National Institute of Optics, National Research Council, Sesto Fiorentino, Italy; 10 Institució Catalana de Recerca i Estudis Avançats (ICREA), Barcelona, Spain; 11 National Center for Radiation Protection and Computational Physics, Istituto Superiore di Sanità (ISS), Rome, Italy

**Keywords:** cerebral cortex, cortical oscillations, slow-wave activity, data analysis, software tools, workflow, reproducibility, validation, wide-field calcium imaging, ECoG

## Abstract

Neuroscience is moving toward a more integrative discipline where understanding brain function requires consolidating the accumulated evidence seen across experiments, species, and measurement techniques. A remaining challenge on that path is integrating such heterogeneous data into analysis workflows such that consistent and comparable conclusions can be distilled as an experimental basis for models and theories. Here, we propose a solution in the context of slow-wave activity (<1 Hz), which occurs during unconscious brain states like sleep and general anesthesia and is observed across diverse experimental approaches. We address the issue of integrating and comparing heterogeneous data by conceptualizing a general pipeline design that is adaptable to a variety of inputs and applications. Furthermore, we present the Collaborative Brain Wave Analysis Pipeline (Cobrawap) as a concrete, reusable software implementation to perform broad, detailed, and rigorous comparisons of slow-wave characteristics across multiple, openly available electrocorticography (ECoG) and calcium imaging datasets.

## Highlights

• A flexible analysis pipeline design enables the integration of heterogeneous data
• The pipeline integrates robust tools and standards
• ECoG and calcium imaging provide complementary but comparable wave characterizations
• The complexity of detected traveling-wave patterns depends on the spatial resolution

## Motivation

Neuroscience is composed of a multitude of domains with specific approaches to measure and analyze neural data. These approaches encompass various temporal and spatial scales, species, and measurement techniques that have traditionally existed in isolation. Only recently has there been a growing recognition of the need to integrate these diverse perspectives. This integration is particularly relevant in computational neuroscience, where the creation of large, biologically realistic models depends upon the availability of comprehensive reference data for calibrating and validating their dynamics. A prominent feature of neural network dynamics is their spatiotemporal organization of activity, such as slow-wave activity (<1 Hz). Slow waves are consistently observed in contexts like anesthesia and NREM sleep across numerous measurement techniques. In this study, we aimed at exploring how an integrative, multi-modal pipeline can serve as a bridge between distinct neuroscience domains encompassing specific measurement types, experimental conditions, and both animal and computational models.

## Graphical abstract

**Figure:**
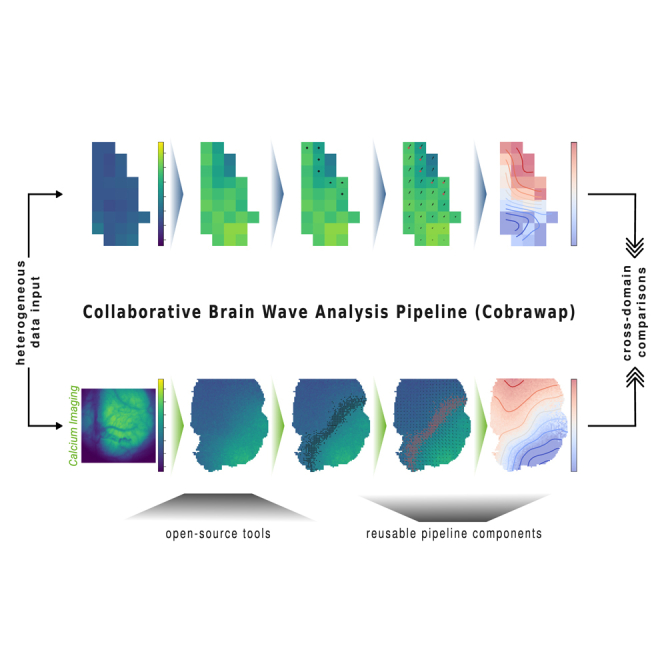

## Teaser

Understanding brain function increasingly requires the aggregation of insights across experiments, measurement techniques, or species. Here, Gutzen et al. demonstrate how an adaptable pipeline design can integrate heterogeneous data into rigorous analysis workflows to distill consistent and comparable conclusions.

## Introduction

Today’s research landscape excels in an unprecedented richness of experimental data and methodologies. In neurophysiology, this enables an array of research applications and a better calibration of models of brain dynamics and function. However, recording techniques differ considerably in the way in which they capture neural activity. These differences^1^^,^^2^ include the type of signal (e.g., electric activity, magnetic fields, fluctuations of calcium concentrations, or radiant isotopes) and the scales on which the signal is observed in terms of temporal resolution (sub-milliseconds to seconds), spatial resolution (micrometer to centimeters), and spatial extent (single electrode to the whole brain). The combination of complementing experimental approaches, each one focusing on specific aspects, enables a more comprehensive understanding of neuronal activity and supports validating findings independently of the biases of individual recording techniques, e.g., spatial vs. temporal resolution, latency, or artifacts.^3^ This raises the challenge to integrate multi-scale, multi-methodology data by defining levels of description and relationships between data modalities.

Cross-domain comparisons between different data modalities form the foundation of validation scenarios that align theories and models with experimental data in the presence of biological variability and data heterogeneity.^4^^,^^5^^,^^6^ However, performing such comparisons is not a trivial task and is only rarely addressed. Even if authors adopt definitions and methods from other publications, comparing quantitative findings is not necessarily straightforward. For example, in the studies Massimini et al.^7^ and Botella-Soler et al.,^8^ the same methodology and wave definition are adopted. Still, a direct quantitative relationship between the reported wave velocities (2.7±0.2 and 1.0±0.2 m/s, respectively) is difficult due to remaining differences in the analysis implementations, making it impossible to accurately retrace the source of the discrepancy. In particular, inaccessible and non-reusable code makes it increasingly ambitious for scientists to interpret and understand the differences in the quantitative results.

The main challenge in cross-domain comparisons is to find a common basis for the analysis. What this constitutes depends on the involved data types and the scientific questions. More similar data can have more immediate commonalities, whereas very different data may only be compared on an abstract level. Generally, the comparison of two datasets benefits from having a common description level for the observations, equivalent or at least comparable methods for processing and analyzing, and the use of equivalent implementations and standard algorithms. Indeed, much care is required to eliminate as many potential confounds as possible, as it has been shown that even seemingly minor influences can have crucial effects on numerical results and add sources of systematic errors.^9^

A prerequisite of comparability is reproducibility. Any analysis result must first be reliably reproducible with the same data before it can be reasonably compared with results from other data. Ideally, the analysis results to be compared are generated from the same code base. However, integrating heterogeneous data, bridging otherwise specific and isolated studies, and contributing to a more collaborative scientific tool base require a considerable degree of generality and reusability of the code. Fortunately, many aspects of analysis workflows are already formalized and addressed by open-source software tools and standards, such as data and metadata representation,^10^^,^^11^^,^^12^ provenance,^13^ version control,^14^ standardized algorithms and frameworks,^15^^,^^16^^,^^17^ and workflow management.^18^^,^^19^^,^^20^

A scenario where a multitude of analysis methods exists, acting on observations from a variety of different measurement techniques, spatiotemporal scales, and species, is the study of spatially organized activity, in particular, wave activity. While waves are a ubiquitous phenomenon, their functional roles are not fully understood.^21^

**Figure 1:**
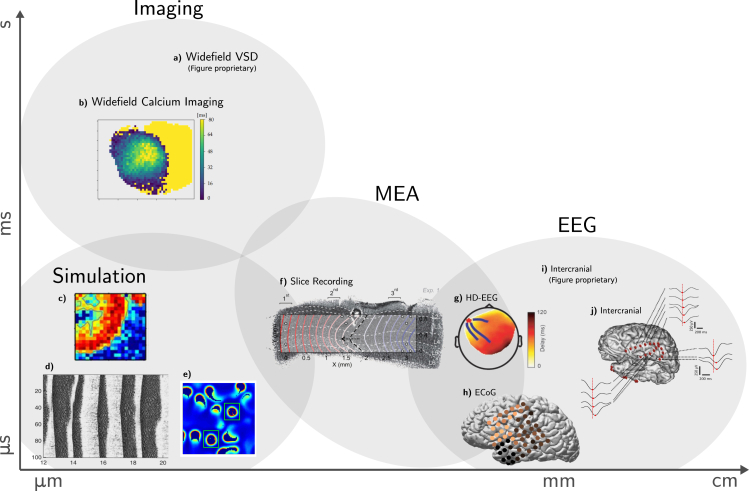
Multi-scale, multi-modality, uniphenomenon: The many faces of slow waves Illustration of examples of slow-wave activity reproduced from the studies cited for each image. (A) Wide-field voltage-sensitive dye imaging of awake mice.^94^ (B) Recorded anesthetized GCaMP6f mice with wide-field fluorescence microscopy.^72^ (C) Distributed network of cortical columns of leaky integrate-and-fire neurons with spike frequency adaptation.^40^ (D) 1D multi-layer thalamo-cortical model with one- and two-compartment neuron models using Hodgkin-Huxley kinetics.^85^ (E) 2D balanced conductance-based spiking neural network model.^43^ (F) Multi-electrode recording in ferret cortical slices.^33^ (G) Human high-density (HD) EEG during first sleep episode of the night.^7^ (H) Human ECoG recording during sleep.^32^ (I) Intracranial depth EEG in sleeping human subjects.^71^ (J) Intracranial depth EEG in humans during sleep.^8^ (B, C, E, H, and J) Licensed under the Creative Commons Attribution 4.0 International License (CC-BY). (D, F, and G) Reproduced with permission. (B, C, E, F, H, and J) Copyright with the respective authors of the cited source publications. (D) Copyright 2002 Society for Neuroscience. (G) Copyright 2004 Society for Neuroscience.

A particular type of wave-like activity that we set out to investigate in this study are slow waves (<1 Hz).^22^ They describe propagating activity patterns in the delta band, defined by transitions between states of low activity (down) and high activity (up). They are reliably observed in mammals during deep sleep and anesthesia (Figure 1) and are frequently investigated in the study of memory, consciousness, and the cognitive effects of sleep.^23^^,^^24^^,^^25^^,^^26^ For slow waves, findings include that the transitions between up and down states are coordinated precisely over a wide cortical range, implying a larger network mechanism.^27^ The transitions coordinate with the synchronization of the astrocytic network,^28^ with thalamic activity,^29^^,^^30^^,^^31^ and across the cortex, as reoccurring slow-wave patterns can appear over an entire hemisphere.^32^ Evidence from slice and *in vivo* recordings further suggests that wave propagation is guided by excitability, i.e., predominantly resides in layers 4 and 5,^33^^,^^34^ and shows distinctly different oscillation characteristics across cortical regions.^35^^,^^36^^,^^37^ Although slow-wave activity is characteristic of sleep and anesthesia, it can even be observed in localized areas during wakefulness.^38^ Additionally, modeling approaches suggest the importance of long-range connections,^39^^,^^40^ synchronous high-amplitude events,^41^ and the correct excitation-inhibition ratio^42^^,^^43^ to exhibit propagating slow waves. In face of such a prevalent phenomenon as slow waves, it is not surprising that the literature reveals a very heterogeneous mosaic of approaches, methods, metrics, and terminology. Due to this plurality, the relationships between the respective findings are rarely apparent and mostly qualitative, limiting the potential of cumulative discovery by the collection of studies. Moreover, it is generally unclear which observables are relevant for the local cortical function or higher cognitive functions (e.g., memory consolidation). The typically reported properties are thus often heuristic and include, for example, transition slopes,^35^ phase velocity,^7^^,^^32^ wave type classification,^44^^,^^45^^,^^46^^,^^47^^,^^48^ source/sink location and propagation patterns,^49^^,^^50^ excitability,^37^^,^^51^^,^^52^ and event frequency.^53^ Thus, we here focus on common observables that can be extracted from different measurement modalities (i.e., planarity, interwave intervals, velocity, and direction). By investigating the relations of these characteristics with parameters such as brain state, anesthetic level, and spatial/temporal resolution, we can evaluate the capabilities of measurement techniques, identify biases, constrain theories, develop and benchmark analysis methods, contribute to defining standards, and aid the assessment of clinical data, for example, in the case of coma patients.

In the following, we report two results: first, we address the technical aspects of developing formalized analysis approaches on a conceptual and an implementation implementation of the Cobrawap level, resulting in the Collaborative Brain Wave Analysis Pipeline (Cobrawap) as a flexible and modular pipeline solution for analyzing cortical slow-wave activity. Second, we demonstrate the application of Cobrawap by performing a structured analysis across multiple heterogeneous datasets dataset comparisons quantify the variability of slow-wave characteristic and a comparison of up-state detection methods by interchanging the corresponding method block interchangeable blocks enable benchmarking of methods. Readers who are more interested in the applicability of the analysis pipeline are invited to skip directly to this second part.

## Results

### A modular analysis approach enables flexibility in studying slow waves

**Figure 2:**
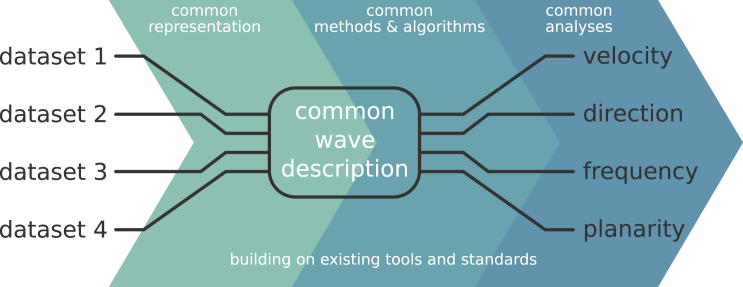
Pipeline approach The proposed pipeline design has the role of aligning methods to create and operate on a common description of the phenomenon of interest. By integrating data from heterogeneous sources on the input side and extracting a variety of common output metrics on the output side, this pipeline approach forms a basis for rigorous comparisons. The pipeline is built on existing tools and standards, e.g., data and metadata representation, file formats, standard packages and implementations, environment handling, and workflow management. The catalog of applicable methods is flexibly extendable, making the analysis pipeline adaptable and reusable.

Since there is no single fully comprehensive measure to characterize spatial activity patterns, we focus on identifying commonly used analysis metrics of wave activity that enable a comparison between datasets of different measurement types. In designing the pipeline, we first align the heterogeneous input data (e.g., from electroencephalogram [EEG], implanted electrode arrays, imaging techniques, or simulations) and find a common representation. Although the input data may differ in terms of spatial or temporal resolution, scale, or signal type, we aim to process them by common methods and to converge toward a common description of wave activity. From this common description, we derive characterization metrics that are agnostic about the data’s origins. Such a generalized approach may be applied to different types of propagating fluctuating activity associated with different brain states, including various expressions of slow waves^54^^,^^55^ (as we do here), burst suppressions,^56^^,^^57^ or waves in higher frequencies.^58^^,^^59^ This way, we arrive at relatable wave characterizations and avoid comparing apples to oranges (Figure 2).

The key to making the pipeline adaptable to the different data processing requirements, analysis approaches, and scientific questions is to identify the appropriate level of modularity. Thus, we first segment the analysis procedure into a series of sequential stages. Each stage is a self-consistent logical step in an analysis workflow with a well-defined purpose, input, and output. A stage should be constructed generally enough to be reusable as a standalone or in other pipelines. Along the pipeline, subsequent stages become necessarily more specific and tailored toward the scientific application, while the early stages cope with more general tasks, such as data integration and preprocessing, that are likely shared across different pipeline applications.

Each stage is further segmented into blocks. A block defines a concrete action to be performed on the data, implementing a method. Similar to stages, blocks have a well-defined input and output by which they can be chained together. In contrast to stages, blocks are not necessarily executed in a predefined sequence. Rather, each stage implements the mechanics of the block interactions and defines which block combinations and sequences can be chosen. Some blocks may need to be mandatory for the realization of the stage purpose and have a fixed place in the execution order. Others may be optional and flexibly combined. We identified three basic arrangements to be employed in the stages: fixed (a fixed execution order of the blocks), choose any (a custom selection and execution order of a set of blocks), and choose one (a multiple-choice selection between blocks for one execution position).

### Implementation of Cobrawap

Based on the conceptual framework illustrated above, we construct the Collaborative Brain Wave Analysis Pipeline (Cobrawap) as a specific pipeline application for the analysis and comparison of slow-wave activity across 5 publicly available datasets of electrocorticography (ECoG) and wide-field calcium imaging recordings of anesthetized mice from the EBRAINS Knowledge Graph platform (https://search.kg.ebrains.eu). Besides the measurement technique, the 60 examined recordings vary in a range of factors such as experimental setup, the genetic strain of the mice, anesthetic type, anesthesia level, temporal and spatial resolution, and recording duration (see experimental model and subject details).

The pipeline implementation uses the open-source language Python to ensure accessibility and reproducibility. Further, we base the pipeline’s architecture on the Python-based workflow manager *snakemake*,^18^ which employs input-to-output rules containing executable shell commands (e.g., Python scripts or bash commands). Snakemake structures the execution of the rules by building dependency trees from the final result file(s) back to the initial input, matching the input requirements to the outputs of preceding rules (see design of the analysis pipeline and Figure S6).

**Figure 3:**
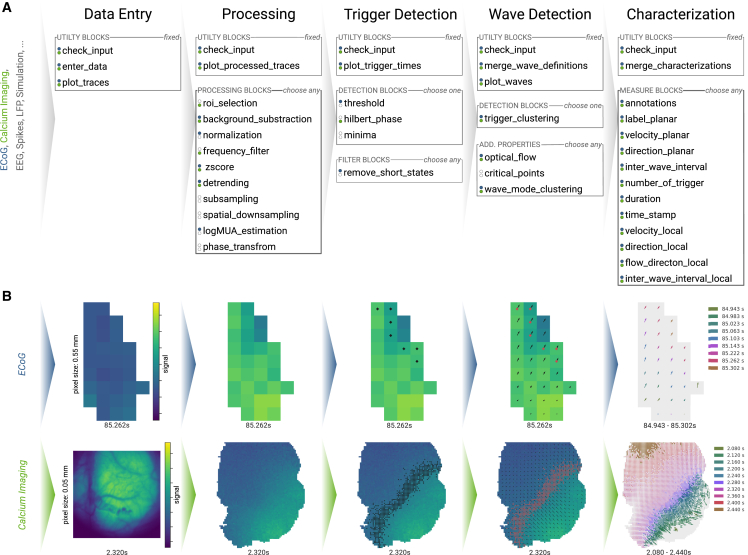
Progression of two datasets, ECoG and wide-field calcium imaging, through the Cobrawap (A) The five successive stages contain collections of modular blocks in three different selection modes (fixed, choose one, choose any). The analysis path is adaptable for specific datasets and analyses by selecting and configuring the desired blocks (indicated by colored dots for the datasets). (B) The intermediate results after each stage are visualized for the two datasets as color-coded signals on the electrode/pixel grid covering most of the right hemisphere of the mouse brain (up: anterior, right: lateral; ECoG: 4.95×2.75 mm, calcium imaging: 5×5 mm). From left to right: raw data, post-processed signal, detected upward transitions (black markers), grouped wavefronts (red markers) with the optical flow (arrows), and quantification of the linear flow alignment within the waves (i.e., planarity).

We organized the Cobrawap into 5 sequential stages, successively transforming the raw data and extracting slow-wave characterizations, as illustrated in Figure 3. In the following, we describe the role of each stage in the analysis of the ECoG and wide-field calcium imaging data. The stages and blocks are described in detail in the STAR Methods and in the corresponding README files.

• In the first stage, data entry, the data are being prepared for the later stages by loading, structuring, and annotating the data and metadata according to the defined scheme using the Neo data representation.^60^ For loading data and converting its often highly specific structure into a common representation, each data source requires a custom script that can be adapted from a template script, making use of Neo for structuring the data and interfacing to a variety of file formats.
• The second stage, processing, offers a series of blocks implementing basic preprocessing steps that can be arbitrarily combined and ordered. The choice of blocks for the two data types is also indicated in Figure 3. The calcium imaging data are cut into a region of interest, the background is subtracted, and the pixel-wise signals are detrended, frequency filtered within 0.1–5 Hz (using a second-order Butterworth filter with the scipy.signal.sosfiltfilt filter function), and *Z* scored. The data from the unfiltered micro-ECoG recordings also have their background subtracted before the electrode-wise signals are detrended, normalized, transformed into a logarithmically scaled multi-unit activity (logMUA) signal with reduced sampling rate (see logMUA estimation (in stage 2)), and *Z* scored.
• The third stage, trigger detection, provides multiple options for a trigger detection method, identifying the time stamps of state transitions (upward or downward trigger) in each channel as an indicator for the possible passage of a wavefront (see trigger detection (in stage 3)). In the following, only upward transitions are considered as triggers. Since the logMUA signal shows sharp state transitions, they are best detected by a threshold determined from a channel-wise fit of the amplitude distributions.^37^ Conversely, the transitions in the imaging data are determined by the slow activation function of the fluorescent indicators. Therefore, they are better detected by identifying the upward slopes by either the Hilbert-phase signal crossing a specific value (here, −π/2) or by the local minima preceding a dominant peak. In the following, we use the trigger detection via the Hilbert phase; however, we later show how interchangeable blocks enable benchmarking of methods.
• In the fourth stage, wave detection, the channel-wise trigger times are grouped to define the individual waves (see trigger clustering (in stage 4)). This wave representation as a collection of local upward transition times is optionally enriched with additional descriptions such as the optical flow estimation (in stage 4) and the critical points of the resulting vector field,^61^ or an additional clustering of the waves into modes, based on the spatial arrangement of the trigger delays.
• The fifth stage, wave characterization, applies a series of quantitative characterizations on the basis of the measures and groupings generated by the previous stages. The selection of characteristics can be tailored toward addressing specific scientific questions or research objectives. To have a consistent output format for this stage, there are two distinct realizations for the fifth stage: one for a characterization using wave-wise measures, e.g., determining one velocity value per wave, and another for a characterization using channel-wise measures, e.g., calculating local velocity values per channel and wave. For simplicity, these two alternatives are presented as a single stage in Figure 3A.

To demonstrate the capabilities of the pipeline approach to generate a meaningful quantification of slow-wave phenomena, we choose four metrics as the basis for dataset comparisons: the local (i.e., channel-wise) interwave interval, velocity, and direction measures and the global (i.e., wave-wise) planarity measure. The interwave interval is defined as the time delay between the occurrence of two consecutive waves at a recording site. The channel-wise velocity, *v*, is calculated from the derivatives of the delay function of a wave, T(x,y), which indicates when a wave has reached the position (x,y) in its propagation^33^^,^^48^^,^^62^:

(Equation 1) $$v_{x,y}=\sqrt{\frac{1}{\partial_{x}T^{2}+\partial_{y}T^{2}}}$$

The channel-wise direction of wave propagation can also be derived from the time-delay function T(x,y). However, in the following, we use the optical flow of the phase signal (see optical flow estimation (in stage 4)). The optical flow is a continuous-vector-valued signal for each position (x,y), indicating in which directions the contour lines of equal phase propagate. We define the channel-wise wave directions of a propagating wave as the optical flow vector directions at the times and positions of its trigger events. The planarity, *P*, of a wave is also defined via the optical flow as the absolute value of the normalized channel-wise direction vectors of all trigger events that belong to a wave, quantifying their alignment on a scale from 0 to 1:

(Equation 2) $$P=\frac{\left\|\sum \vec{v_{i}}\right\|}{\sum \left\|\vec{v_{i}}\right\|}$$

**Figure 4:**
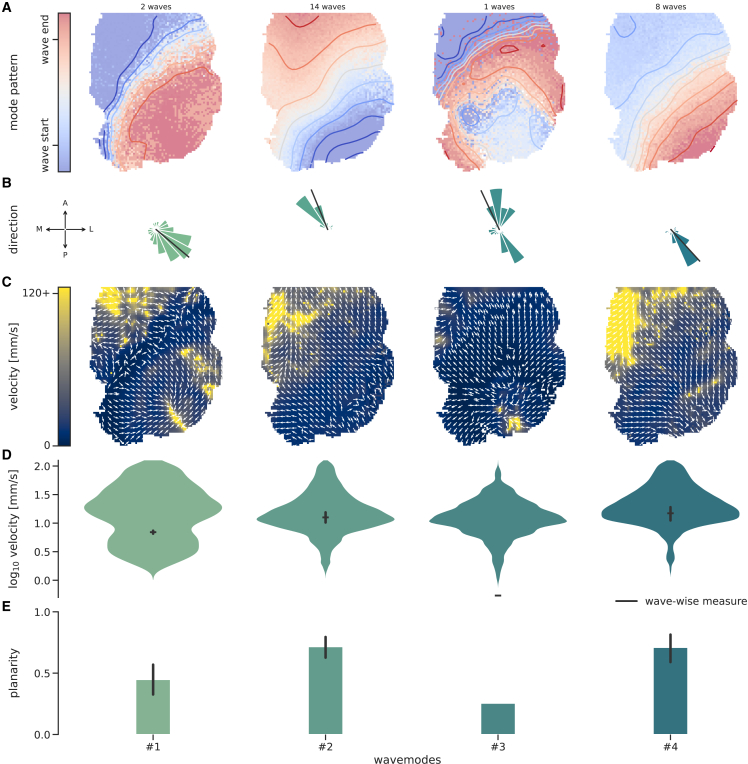
Representation of the Cobrawap output for one exemplary wide-field calcium imaging recording The waves are categorized into one of 4 modes (block wave_mode_clustering), shown in columns. (A) The average wave pattern of each mode is illustrated as a time-delay heatmap with iso-delay contours. Top: number of waves contributing to the mode. (B) The aggregated histograms of channel-wise directions during waves of each mode. The black lines indicate the average wave-wise direction measures. (C) Map of the average channel-wise velocities $v_{x,y}$ in waves of each mode (color code), overlayed (arrows) with the average channel-wise direction determined via the optical flow. (D) The distributions of channel-wise velocities corresponding to (C). Black ticks and error bars: the average and 95% confidence interval (CI) of the corresponding wave-wise velocities. (E) The average and 95% CI of the planarity, *P*, for waves of each mode.

Figures S1 and S2 show videos of the wave activity (stage 4 output) for two example recordings. The pipeline output (of stage 5) is a table of the characteristic measures derived from the detected wave activity. Figure 4 presents some of the output measures for one of the calcium imaging recordings. An analogous example figure for an ECoG recording is shown in Figure S3. The channel-wise and wave-wise direction and velocity, as well as the wave-wise planarity, are summarized for 4 groups of similar waves, i.e., “wave modes.” The wave-mode clustering method (implemented as an optional block in stage 4 of the pipeline) applies a k-means clustering on the trigger delay matrix containing the relative trigger times for each channel in each wave.^33^^,^^35^^,^^48^ The number of modes was set by hand to reasonably represent the variability of wave types in the recording. Generally, the “optimal” number of modes to set for the k-means algorithm depends on recording and the analysis application.

For the presented recording, most waves are relatively planar and travel along the lateral posterior-to-medial anterior axis (modes #2 and #4). Mode #1 is a variation of mode #4 with a lower average velocity, and mode #3 contains only one wave. Although the channel-wise and wave-wise measures for the direction and velocity (Figures 4B and 4D) are defined differently, they agree considerably well for modes #1, #2, and #4 when the wave pattern is predominantly planar, while a single wave-wise value cannot accurately capture more complex wave patterns (i.e., mode #3). Otherwise, the different measures provide a coherent characterization of each wave mode. In the following, we only consider the channel-wise measures, with the exception of the wave-wise planarity measure *P*, which has no channel-wise equivalent.

### Dataset comparisons quantify the variability of slow-wave characteristics

Based on the Cobrawap implementation, we are now in a position to perform quantitative comparisons of slow-wave dynamics across the described ECoG and wide-field calcium imaging datasets, contrasting various experimental parameters. In the following, we demonstrate the application of the pipeline to investigate the influences of the anesthetic type and dosage, the application of disease models via genetic knockout, and the measurement technique itself, in particular its spatial resolution.

**Figure 5:**
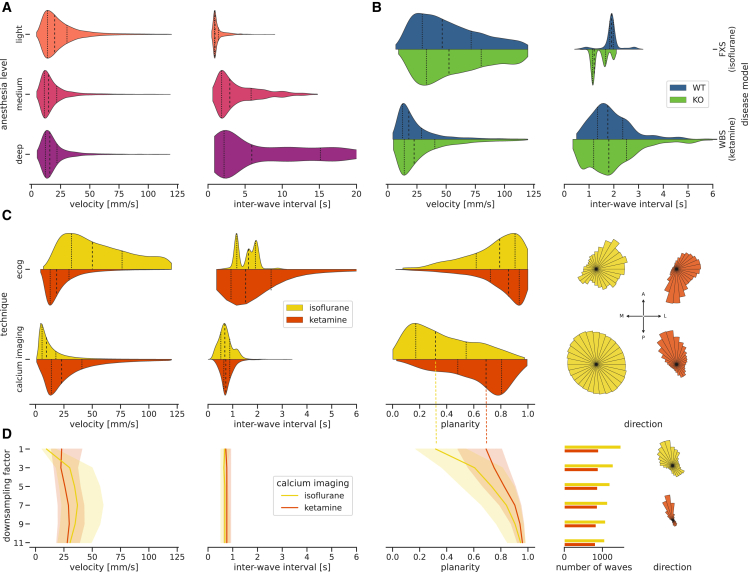
Quantitative comparison of slow waves across heterogeneous datasets Violin plots show sample distributions with indications of the median (dashed line) and the quartiles (dotted lines). Line plots also show the median and quartiles (shaded areas). Polar plots show the distributions of wave directions in the right hemisphere so that “up” corresponds to an anterior direction and “right” to a lateral direction. (A) Velocity and interwave intervals of slow waves in ECoG recordings as a function of the anesthesia level. (B) Velocity and interwave intervals of slow waves in ECoG recordings of experiments modeling Willems-Beuren syndrome (WBS) and Fragile X syndrome (FXS) split into wild-type (WT; blue) and knockout (KO; green) subjects. (C) The ECoG data from (B) is compared to calcium imaging data split into anesthetic types, on the basis of wave velocity, interwave interval, wave planarity, and wave direction. All distributions in (A)–(C) are scaled to have equal height. (D) Effect of stepwise spatially downsampling the calcium imaging data from 0.05 (factor 1) to 0.55 mm (factor 11; the spatial resolution of the ECoG data) on wave velocity, interwave interval, wave planarity, number of waves, and wave direction. The line graphs denotes the median and the shaded area the 0.25–0.75 quantile of the corresponding distributions. The histograms of wave directions are only shown for the fully downsampled data (with factor 11).

As shown by Ní Mhuircheartaigh et al.,^63^ the slow-wave activity power increases with anesthetic concentration until reaching saturation. In preliminary analyses, we examined this relation and ensured that the pipeline methods are robust to reliably process activity recordings from different anesthesia levels (Figure S4). To further check the validity of the pipeline, we qualitatively replicate results that were previously published using the same datasets. While the velocity of waves tends to decrease slightly in deeper anesthesia states,^48^ the interwave intervals become more prolonged, i.e., the frequency of waves decreases.^48^^,^^64^ The same trends are visible in the corresponding pipeline output for the same data (Figure 5A). The velocity and frequency of slow waves were also measured in the context of a disease model for Williams-Beuren syndrome (WBS) in knockout (KO) and wild-type (WT) conditions (of the same genetic strain).^65^^,^^66^ In both the previous publication and the pipeline output (Figure 5B), we observe no visible effect on the wave characteristics except for a slight increase in the variance in the KO condition.

Including another dataset from an experiment^67^ that models the Fragile X syndrome (FXS) allows us to extend the analysis of the WBS data across experiments. Focusing on the WT control subjects, we compare the influence of experimental parameters between the WBS and FXS experiments (Figure 5B). A notable difference between the two experimental setups is that the WBS experiment used ketamine as the anesthetic (100 mg/kg inducing +37 mg/kg maintaining), while the FXS experiment used isoflurane (4% inducing +1% maintaining). Comparing the measure distributions for the WT mice shows considerably larger velocities measured in the experiment that used isoflurane and a larger range of interwave intervals for the experiment that used ketamine (Figure 5B). In comparison to Figure 5A, where anesthesia was induced with ketamine (75 mg/kg) but maintained with isoflurane (0.1%–1.16%), we see a better agreement with the velocities in the WBS (ketamine) experiment than with the FXS (isoflurane) experiment. However, comparing the exact depth of anesthesia across different anesthetics is generally difficult. Furthermore, it is to be noted that since this is a meta-analysis, there is little control for confounding parameters between the different datasets, so care must be taken in the attribution of the differences in wave characteristics to a single parameter, here the anesthetic type.

Next, we broadened the scope of the analysis by contrasting the ECoG recordings of ketamine- and isoflurane-anesthetized mice with analogous recordings that use wide-field calcium imaging on anesthetized Thy1-GCaMP6f mice, measuring the cortical activity via the fluorescent response in excitatory neurons.^68^^,^^69^ Figure 5C illustrates the distributions of wave characteristics grouped by measurement technique and anesthetic type. A principal difference between the measurement techniques is their spatial resolution. The wide-field calcium imaging data have a resolution of 0.05 mm compared to 0.55 mm for the ECoG data. The finer resolution allows for a better distinction of complex non-planar wave patterns, as can be seen by the broader distribution of the planarity that is shifted toward smaller values. Additionally, in calcium imaging data, complex wave patterns with low planarity are more prevalent under isoflurane-induced anesthesia than under ketamine-induced anesthesia, an effect that can also be seen to a smaller extent in the ECoG recordings. Furthermore, the detected waves in the calcium imaging data are more frequent and regular, as shown by the interwave-interval distributions. The wave velocity distributions exhibit a notable discrepancy between the measurement techniques for the isoflurane datasets, while the velocities for the ketamine dataset are quite similar. This considerable difference in wave velocities is likely related to a difference in the isoflurane concentration (1% in ECoG and 1.5%–2% in calcium imaging recordings), as even small differences in the concentration can have a considerable effect on the wave dynamics (cf. Figures 5A and 5B).

The slow waves we detect with the Cobrawap tend to propagate along a preferred axis and primarily in one direction. This axis seems to be approximately consistent within the data of each measurement technique but not across (Figure 5C, right). In the ECoG data, the preferred propagation axis spans from posterior medial to anterior lateral, with the preferred directions being different for the isoflurane and ketamine datasets. In the wide-field calcium imaging data, the preferred wave direction is from posterior lateral to anterior medial. Wave propagation that is oriented in a back-to-front or front-to-back manner is also reported in previous studies.^7^^,^^31^^,^^35^^,^^48^^,^^62^^,^^70^^,^^71^ The spread of the wave direction histogram around the preferred directions can be either caused by a variance of channel-wise directions between waves or within waves, e.g., waves with low planarity have, per definition, a broader spread of channel-wise directions.

To further explain the observed differences in the wave characteristics between the ECoG and calcium imaging data, we investigate the influence of their different spatial resolutions by spatially downsampling the calcium imaging data up to a factor of 11, for which the spatial resolution is equal to the one of ECoG (0.55 mm). Figure 5D shows how the distributions of wave characteristics change as a function of the downsampling factor. With a decreasing spatial resolution, fewer waves are detected, and they appear more planar, as some complex local patterns are no longer detected. This effect is particularly visible for the isoflurane datasets. A similar effect on the probability of detecting a planar wave as a function of region of interest size has been previously shown by Liang et al.^50^ The histograms of directions of the fully downsampled calcium imaging data are more narrow than for the full resolution (Figure 5C), indicating that the propagation directions are consistent across waves, and the variances in direction observed in Figure 5C are caused mainly by non-planar waves. Lastly, we observe that with downsampling, the waves in the isoflurane datasets exhibit faster channel-wise velocities that surpass the ketamine wave velocities, comparable to the ECoG data.

In summary, we demonstrate how the adaptable pipeline approach of Cobrawap enables the comparison of slow-wave characteristics across heterogeneous datasets, including electrical and optical acquisition methods. This meta-analysis illustrates distinct differences within the aggregated data and potential dependencies on the experimental parameters to be investigated further.

### Interchangeable blocks enable benchmarking of methods

**Figure 6:**
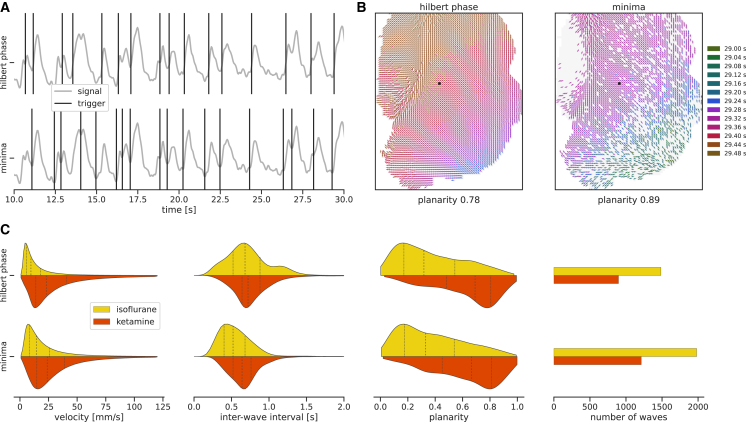
Comparison of trigger detection methods in calcium imaging data (A) Upward transitions (triggers, black vertical lines) found by two different detection algorithms, detecting the crossing of the Hilbert phase at −π/2 (top) and local minima (bottom). Signal taken from the black pixel indicated in (B). (B) The same exemplary wave (corresponding to the last trigger in A) illustrated over the recorded area as detected using the two trigger detection algorithms. The arrows indicate the local direction of the wavefront at the time of the trigger, which is also encoded as the color of the arrows. (C) The distributions of the wave velocity, interwave interval, wave planarity, and number of the waves obtained by the two methods in the calcium imaging datasets (compare to Figure 5C). All distributions in (C) are scaled to have equal height.

While applying the same analysis method to different data enables rigorous comparisons, applying alternative methods to the same data allows investigating the influence of the choice of the method itself. In the analysis of slow waves, the method for detecting the transitions from down to up states plays a central role that we will consider as an example in the following. So far, we detected the trigger times in the calcium imaging data at the upstroke of the transitions, with the Hilbert phase of the signal crossing a threshold value of $-\frac{\pi }{2}$ (see trigger detection (in stage 3)). However, alternative methods to define trigger times were suggested, such as using the local minima of the filtered signal.^72^ Figure 6 illustrates the influence of the two different detection methods on the resulting wave characteristics. In Cobrawap, realizing this method benchmarking workflow only requires selecting the corresponding wave detection block and rerunning the analysis on the calcium imaging data. The detected triggers differ clearly in number and exact timing (Figure 6A), resulting in a different set of detected waves (see an example in Figure 6B). Figure 6C shows that the total number of waves is larger with the minima method ensuing that the corresponding interwave intervals also tend to be shorter than for the Hilbert-phase method (effect sizes: 0.43 for ketamine and 0.58 for isoflurane). The velocities remain similar for the ketamine datasets (effect size: 0.04) but differ slightly for the isoflurane datasets (effect size: 0.32), while the planarity distributions are mostly unaffected by the choice of trigger detection method (effect sizes: 0.03 for ketamine and 0.02 for isoflurane). A Kolmogorov-Smirnov test indicates significant (p<0.01) differences for the velocity and interwave interval but not for the planarity.

The ability to easily compare methods allows us to evaluate the strengths of each approach and check for potential biases introduced to the wave characterization. The Hilbert-phase method employs a non-parametric approach (using only an arbitrary phase threshold value), detecting two succeeding local peaks as separate triggers only when the corresponding phase of the signal completed a full rotation between them. Therefore, the method is more conservative and may disregard some overlapping slow-wave activity peaks as “noise” fluctuations (cf. third to last trigger in the top panel of Figure 6A). While this results in fewer waves, they are, however, better separated and more coherent across channels, whereas the minima detection method uses multiple parameters to fine-tune the algorithm to the researcher’s expectation of which minima should constitute a slow-wave trigger in the respective dataset. While such method calibration offers flexibility, it can be very intricate, especially when handling multiple large datasets where a common parameter selection for comparability is an additional concern. Here, this results in a less strict selection of slow-wave triggers. For a more extensive method comparison, including specific edge cases, this approach could be further combined with simulated data.

## Discussion

### Advantages of a reusable modular pipeline design

The presented multi-modal analyses of slow-wave activity using the Cobrawap implementation illustrate the benefits of a modular pipeline approach that incorporates general aspects of reproducibility and reusability. The pipeline output retains information about the applied analysis scripts, their execution order, and parameter settings. The intermediate stage and block results and their visualizations further help to retrace the workflow and build confidence in the findings. Aligning the workflows for different datasets by applying the same or analogous analysis methods while catering to their specific processing demands makes the corresponding results comparable. This setup promotes cross-domain comparisons, including the quantitative evaluation of experimental parameters (e.g., measurement techniques, anesthetics, species) and validation of simulated activity data. The modular nature of the pipeline design can cater to heterogeneous data inputs. Additionally, interchanging methods in the analysis of the same dataset also allows the evaluation of a method’s influence on the downstream results. The explicit extensibility of the pipeline and reusability of the individual components aim to facilitate further research applications by providing a framework for designing efficient and reproducible workflows. Still, care has to be taken to adequately match the requirements of data and methods. For example, the “threshold” and Hilbert-phase trigger detection methods assume a channel-wise quasi-stationarity of the down- and up-state levels after the processing stage (including, e.g., detrending). Non-stationary recordings (e.g., due to changing anesthetic concentration or wave frequency regime) should therefore be cut into quasi-stationary segments or analyzed with correspondingly adaptive methods (like the minima trigger detection).

### Structured analysis pipelines contribute to progressing the study of slow waves

All measurement techniques have a bias in terms of which aspect is recorded from a neural population. This results in different compositions of subsampled neural activity. In the presented meta-analysis, we compare the neural activity characteristics represented in ECoG and wide-field calcium imaging recordings (Figure 5). These measurement techniques produce fundamentally different perspectives of the underlying activity. On the one hand, ECoG records the spiking activity of neurons in the superficial layer that have a high firing rate and a high signal-to-noise ratio with high temporal but low spatial resolution. On the other hand, wide-field imaging of GCaMP6f in Thy1-GCaMP6f mice measures population spiking activity from excitatory neurons in layers 2/3 and 5 as a delayed, low-pass-filtered, non-linearly transformed fluorescence signal with low temporal but high spatial resolution.^73^^,^^74^^,^^75^ These two measurement perspectives are complementary, as prior work shows that even elaborate models cannot capture their complex relationship, and there is generally no precise agreement between the observations.^76^^,^^77^^,^^78^ Therefore, depending on the focus of the wave activity analysis, both techniques offer advantages. ECoG is better suited to explore the fine temporal dynamics (e.g., the exact shape of up transitions), while calcium imaging can better resolve the intricate spatial dynamics (e.g., complex local propagation patterns). Combining measurements of different spatial resolutions and scope allows us to study the interaction of wave dynamics across scales. For example, the combination of different measurements that sample from different cortical layers can support more detailed investigations of layer-specific contributions to the wave activity. This is of particular interest since aspects like frequency power, signal flow, and propagation speed are known to vary considerably with cortical depth.^33^^,^^79^^,^^80^^,^^81^ Furthermore, since researchers or clinicians might be constrained to a particular measurement setup, in order to provide context, it becomes crucial to understand the extent to which their observations can be related with observations obtained with other measurement techniques or subjects or on different scales. Establishing such links requires integrative analysis approaches, such as the one we propose with Cobrawap, that can combine data from heterogeneous sources.

Besides the biases of the measurement technique and its resolution, we further present the influences of the anesthetic type and dosage on the wave characteristics, showing in particular that ketamine tends to produce more planar waves than isoflurane, in turn also influencing the measured directions and velocities. This effect is likely linked to the known attributes of the anesthetics: that ketamine is more effective in generating slow-wave activity as it increases the power of the local field potential (LFP) in the delta frequency band, while isoflurane rather enhances LFP activity in the theta band and above.^82^^,^^83^

The need to quantitatively relate results from the literature to each other becomes quite apparent when investigating the sources of variance of the velocity of slow waves, which can vary from a few mm/s in recordings of anesthetized rodents up to ∼10 m/s in human sleep experiments.^7^^,^^32^^,^^35^ Studied influences to this variability include the extent of axonal projections,^7^^,^^39^^,^^84^ axonal conductances,^35^ involved cell types,^85^ and neuronal excitability depending on anesthetics,^48^ neuromodulators,^86^ or cortico-cortical or cortico-thalamic loops.^70^^,^^87^ Furthermore, the velocity of a wave may depend on its direction, which in turn is influenced by an interplay of local and global connectivity properties and frequency effects.^7^^,^^87^^,^^88^ Comparison between data from different studies can help relate and discern such influences.

### Integration in model development and data-driven simulations

While the exploration of wave characteristics under different conditions can provide further insight into the understanding of the underlying processes, availability of experimental data can also suffer from constraints in the data size, parameter regime, and uncontrolled confounds. Therefore, in many scenarios, it is beneficial to include modeling data in the analysis. Cobrawap can be directly applied to simulation outcomes to extract the same characteristics as from the experimental data and to perform a quantitative comparison in a subsequent validation step. Such a step can already be integrated into the model development in the form of an explicit calibration. This strategy is considered by Capone et al.,^89^ where, after a preliminary estimation of model parameters through likelihood maximization,^90^ a subset of parameters is further adjusted by performing a grid exploration relying on the direct comparison between data and simulations based on Cobrawap. The comparisons in Capone et al.^89^ are derived from the selection of observables described here, considering the waves’ local velocities, directions, and frequencies. This calibration approach allowed for a meta-inference procedure, finding the optimal parameters of a neuromodulation current to reproduce the dynamics observed in experimental data. Such an approach is essential to complement the theoretical understanding of the relationship between the spatiotemporal features of cortical waves and the cortical structure.^91^

### Reusability: Related pipelines and outlook

We developed the Cobrawap to be reusable. Its modular structure of stages and blocks allows for reuse in different scenarios. The pipeline may be applied to other types of input data, extended by other method blocks, or changed to produce additional kinds of output. The pipeline can be adapted in this regard by editing the stage’s config files and changing the block selection and parameter settings. The minimum requirement for any input data is that they are recorded on a grid electrode/pixel layout. Cobrawap can be extended for more substantial changes by adding new blocks that implement specific analysis methods. Further, disparate applications may swap out the later stages of the pipeline entirely, i.e., realizing a branching-off pipeline (similar to the separate stage 5 realizations for channel-wise and wave-wise observables). The individual blocks and stages can also be used selectively as standalone elements without the pipeline in different workflow applications.

For example, current work entails a more detailed analysis of the local oscillations ignoring the spatial propagation, similar to work done in De Bonis et al.^37^ This application reuses the first three stages of Cobrawap and then branches off with specialized stages. There are also wave-like phenomena in other frequency regimes. For example, in alpha, beta, and gamma frequency ranges, diverse wave patterns have been observed in awake, behaving animals.^45^^,^^58^^,^^61^^,^^92^^,^^93^ A flexible pipeline approach following Cobrawap may disentangle some of the reported results, methods, and terminologies.

### Conclusion

In this article, we demonstrate the advantages of formalizing and harmonizing analysis approaches. By taking a data-science perspective, we work toward integrating heterogeneous insights from different data and analysis types. In our view, understanding an organ as complex as the brain requires the integration of data obtained on multiple levels of observation. Furthermore, we experienced how structuring our methodology and implementation also contributed greatly to our structure of thought. We are confident that the concepts presented in the framework of the Cobrawap implementation contribute to advocating for the concept of reusability for analysis resources, in particular with regard to the uptake of and contribution to community software projects.

### Limitations of the study

The Cobrawap framework is designed to be adaptable and extendable to fit a diverse range of data sources. Nevertheless, its applicability requires certain minimal prerequisites to be met. Currently, the pipeline ingests activity data recorded with a spatial 2D arrangement of simultaneous recording sites, such as electrodes or pixels. Users must exercise care and knowledge in selecting appropriate method blocks for their datasets and should consider data-specific processing requirements, accounting for factors such as non-stationarity. The pipeline implementation allows users to add specific blocks or stages when necessary for particular applications. While Cobrawap aims at accelerating the analysis of various data types and providing a common basis for their comparison, different experimental data sources may inherently contain uncontrolled confounding factors. These need to be taken into account when interpreting the results obtained with Cobrawap. The characterization of modeled wave activity offered by the pipeline can equally serve as a foundation for calibration and validation processes during model development and refinement. There are further ongoing developments to address the described limitations.

## STAR★Methods

### Key resources table

**Table:** 

REAGENT or RESOURCE	SOURCE	IDENTIFIER
**Deposited data**		

Mouse ECoG (WBS)	EBRAINS KnowledgeGraph	https://doi.org/10.25493/DZWT-1T8
Mouse ECoG (FXS)	EBRAINS KnowledgeGraph	https://doi.org/10.25493/ANF9-EG3
Mouse ECoG (Propagation modes)	EBRAINS KnowledgeGraph	https://doi.org/10.25493/WKA8-Q4T
Wide-field calcium imaging (ketamine)	EBRAINS KnowledgeGraph	https://doi.org/10.25493/QFZK-FXS
Wide-field calcium imaging (isoflurane)	EBRAINS KnowledgeGraph	https://doi.org/10.25493/XJR8-QCA

**Experimental models: Organisms/strains**		

Mus Musculus: C57BL/6J	IDIBAPS	RRID:IMSR_JAX:000664
Mus Musculus: Del(5Gtf2i-Fkbp6)1Vcam/Vcam (WBS-KO)	IDIBAPS	N/A
Mus Musculus: ATJ/FVB.129P2-FMR1-mix (FXS-KO)	IDIBAPS	N/A
Mus Musculus: C57BL/6J-Tg(Thy1-GCaMP6f)GP5.17Dkim/J	LENS	RRID:IMSR_JAX:000664

**Software and algorithms**		

Analysis and plotting scripts	This paper	https://doi.org/10.5281/zenodo.10210141
Collaborative Brain Wave Analysis Pipeline (Cobrawap)	This paper	https://doi.org/10.5281/zenodo.10198748 RRID:SCR_022966
Snakemake	Mölder et al. ^18^	RRID:SCR_003475
*Neo*	Garcia et al. ^60^	RRID:SCR_000634
Nix	Stoewer et al. ^97^	RRID:SCR_016196
Elephant	Denker et al. ^16^	RRID:SCR_003833
Scipy	Virtanen et al. ^15^	RRID:SCR_008058
Pandas	McKinney ^96^	RRID:SCR_018214

### Resource availability

#### Lead contact

Further information and requests for resources should be directed to and will be fulfilled by the lead contact, Robin Gutzen (r.gutzen@fz-juelich.de, OrcID: 0000-0001-7373-5962).

#### Materials availability

This study did not generate new unique reagents.

#### Data and code availability

• This paper analyzes existing data that is publicly available via the EBRAINS Knowledge Graph. The DOIs are listed in the key resources table.
• All original code has been deposited at Zenodo and is publicly available as of the date of publication. The DOIs are listed in the key resources table.
• Any additional information required to reanalyze the data reported in this paper is available from the lead contact upon request.

### Experimental model and subject details

An overview of all the individual recordings is presented in Figure S5.

#### Mouse ECoG recordings

The three experimental ECoG datasets have been provided by IDIBAPS (Institut d’Investigacions Biomèdiques Agustí Pi i Sunyer): Williams Beuren Syndrome (WBS) 3–4 months old adult male mice (Wild-Type and Knock-Out), Fragile X Syndrome (FXS) (Wild-Type and Knock-Out) mice and Propagation Modes of Cortical Slow Waves across anesthesia levels in adult male C57BL/6J mice (PMSW). All animals were bred in-house at the University of Barcelona and kept under a 12 h light/dark cycle with food and water *ad libitum*. All procedures were approved by the Ethics Committee at the Hospital Clínic of Barcelona and were carried out to the standards laid down in Spanish regulatory laws (BOE-A-2013-6271) and European Communities Directive (2010/63/EU).

For WBS subjects, an intraperitoneal injection of ketamine (100 mg/kg) and medetomidine (1.3 mg/kg) was administered to induce anesthesia. It was maintained by a constant administration of subcutaneous ketamine (37 mg/kg/h). For FXS subjects, anesthesia was induced by the inhalation of 4% isofluorane in 100% oxygen for induction and 1% for maintenance. Finally, for PMSW subjects, an intraperitoneal injection of ketamine (75 mg/kg) and medetomidine (1.3 mg/kg) and maintained by the inhalation of different concentrations of isoflurane in pure oxygen. In PMSW, three levels of anesthesia were reached that were classified according to the provided isoflurane concentrations: deep = 1.16±0.08% (s.e.m); medium = 0.34±0.06%; light = 0.1±0.0%. The volume delivered was 0.8 L/min.

In order to avoid respiratory secretions and edema, atropine (0.3 mg/kg), methylprednisolone (30 mg/kg), and mannitol (0.5 g/kg) were administered subcutaneously to all subjects. So as to aid breathing and once in the surgical plane of anesthesia, a tracheotomy was performed. The animal was then placed on a stereotaxic frame (SR-6M, Narishige, Japan) with constant body temperature monitoring maintained at $37^{\circ }$ C by means of a thermal blanket (RWD Life Science, China). A wide craniotomy and durotomy were performed over the left or right (only left in FXS) hemisphere from −3.0 mm to +3.0 mm relative to the bregma and +3.0 mm relative to the midline. A 32-channel multielectrode array (550μ m spacing, 50μ m electrode diameter) covering a large part of the hemisphere’s surface was used to record the extracellular micro-electrocorticogram (micro-ECoG) activity. For WBS and FXS datasets, recordings were acquired from spontaneous activity in the animal under anesthesia. Regarding the PMSW dataset, each anesthesia level was maintained for 20–30 min, and spontaneous recordings were consistently obtained in a stable slow oscillatory regime (approximately 10 min after the change in concentration). During the recording protocol, a precise visual inspection of all channels was made in order to ensure that all of them were properly acquiring the signal.

The signals were amplified (Multichannel Systems, GmbH), digitized at 5 kHz, and fed into a computer via a digitizer interface (CED 1401 and Spike2 software, Cambridge Electronic Design, UK).

#### Mouse wide-field calcium imaging recordings

Experimental data acquired from mice have been provided by LENS, European Laboratory for Non-Linear Spectroscopy (http://www.lens.unifi.it), and by the Department of Physics and Astronomy of the University of Florence. All procedures involving mice were performed in accordance with the rules of the Italian Minister of Health (Protocol Number 183/2016-PR). Mice were housed in clear plastic enriched cages under a 12 h light/dark cycle and were given *ad libitum* access to water and food.

Mouse Model: The transgenic mouse line used is the C57BL/6J-Tg(Thy1GCaMP6f)GP5.17Dkim/J, referred to as GCaMP6f mice, from Jackson Laboratories (Bar Harbor, Maine USA) (for more details, see The Jackson Laboratory, Thy1-GCaMP6f, https://www.jax.org/strain/025393). In this mouse model, the ultra-sensitive calcium indicator (GCaMP6f) is selectively expressed in excitatory neurons.^76^^,^^95^

Surgery and wide-field imaging: Surgery procedures and imaging protocols were performed as described in.^72^ Briefly, 6 months old male mice are anesthetized with either a mix of ketamine and Xylazine in doses of 100 mg/kg and 10 mg/kg respectively or isoflurane (3−4% induction and 1.5−2% maintaining). To obtain optical access to neuronal activity over the right hemisphere, the local anesthetic lidocaine (20 mg/mL) was applied and the skin and the periosteum over the skull were removed. Wide-field imaging was performed right after the surgical procedure. GCaMP6f fluorescence imaging was performed with a 505 nm LED light (M505L3 Thorlabs, New Jersey, United States) deflected by a dichroic filter (DC FF 495-DI02 Semrock, Rochester, New York, USA) on the objective (2.5x EC Plan Neofluar, NA 0.085, Carl Zeiss Microscopy, Oberkochen, Germany). The fluorescence signal was selected by a band-pass filter (525/50 Semrock, Rochester, New York, USA) and collected on the sensor of a high-speed complementary metal-oxide semiconductor (CMOS) camera (Orca Flash 4.0 Hamamatsu Photonics, NJ, USA).

### Method details

#### Design of the analysis pipeline

##### Code development

The implementation of the “Collaborative Brain Wave Analysis pipeline” (Cobrawap) infrastructure is being developed on GitHub (https://github.com/NeuralEnsemble/cobrawap) and the corresponding documentation is found on readthedocs (https://cobrawap.readthedocs.io) The pipeline configuration for the presented pipeline application and additional analysis and plotting code is stored in a separate repository (https://gin.g-node.org/INM-6/cobrawap_publication_code).

##### Terminology

We organize the analysis pipeline hierarchically into three layers. The top layer constitutes the pipeline itself or a task-specific realization of a pipeline, which we here call *workflow*. A *pipeline* we define as a sequence of processing/analysis stages to be executed following a given order (“from left to right”). As a *stage* we describe a self-consistent logical episode within the analysis process, such that the output of a stage can be considered a reasonable intermediate result. Furthermore, a stage should be general enough to be reusable in multiple workflows or pipelines. Each stage is segmented into blocks, which can be selected and rearranged depending on the configurations of the user and the mechanics of the stage. A *block* is the smallest unit of the analysis pipeline and performs a specific action on the data. Blocks implement methods. In the case of alternative methods or alternative algorithms implementing a method, they can be either represented as options of a single block or separate blocks.

##### Implementation with snakemake

We designed the structure of the pipelines having in mind the features of the Snakemake workflow management framework.^18^ The rules are defined in script files called S*nakefile* which also link to a config file. Thus, our pipeline structure is conveniently mappable onto the snakemake elements: blocks are represented by rules and stages by Snakefiles. In addition, we use another pipeline Snakefile to combine the stages as snakemake *subworkflows* and make the pipeline executable as a whole. Figure S6 illustrates the blocks in each Snakefile and their execution orders for the two example datasets. Within the stage Snakefiles, each block is represented by a snakemake rule which in most cases executes a Python script. Furthermore, we expand the standard functionality of snakemake by three mechanics required by our pipeline design: 1) chaining the stages by linking the outputs and inputs of subworkflows, 2) manually selecting a specific block (i.e., method) or a sequence of blocks by choosing the desired methods in a config file, and 3) selecting and switching between sets of configs files (*”profiles”*) for all stages.

##### Modularity

One of the main design principles in constructing the analysis pipeline is modularity. This has the purpose of making the pipeline flexible and thus adaptable to different demands, by making it possible to rearrange and switch elements of the pipeline. In contrast to other typical analysis workflows, here, the construction of a specific workflow does not require the changing of any scripts but is rather like tracing a path along the selected stages and blocks within a larger framework offered by the pipeline. Practically, for the stages, this means that different combinations or variations of stages can be chained together. For the selection blocks, there are two flavors of modularity used in the stages: *choose one*, selecting one method block from multiple options; and *choose any*, selecting any number of method blocks in any order (see Figure 3). Another aspect of modularity is that each element should be usable on its own as well as in combination with other elements. Therefore, much care needs to be put into managing the respective interfaces where the elements interact.

##### Pipeline stages

For the analysis of slow wave activity, we chose five stages (Figure 3) starting from more generic stages (Data Entry, Processing) to task-specific stages (Trigger Detection, Wave Detection, Wave Characterization) which build up the Collaborative Brain Wave Analysis Pipeline (Cobrawap).

1 **Data Entry**: This first stage loads a dataset and the required and optional metadata and converts the data into a standardized representation scheme (using the Neo data format). This loading script is the only custom code that is required to add a new data source to the pipeline, integrating information from a data file and a corresponding config file. It is checked whether the resulting data object conforms with the requirements of the pipeline and an overview of a data sample is plotted.
2 **Processing:** In the second stage, the data is prepared for analysis. The user can select any combination of processing blocks to fit the data type and their analysis objectives. Where available, the blocks use standard function implementations by the Elephant Electrophysiology Analysis Toolkit,^16^ the stack of scientific Python packages (i.e., scipy, scikit, etc.), or algorithms from the literature.
3 **Trigger Detection:** Based on the processed data, this stage detects the transition times from Down to Up states (upward transitions, i.e., trigger) and, if possible, Up to Down states (downward transitions) by applying one of the available trigger detection blocks. What this trigger exactly relates to depends on the dataset, the processing, and the detection method. Additionally, there are optional filter blocks that can be applied to clean the collection of detected triggers. The trigger collection is added as a neo.Event named ’transitions’ to the input *Neo* object containing the processed data. This stage is general enough to also be of use for the analysis of other wave-like activity, beyond slow waves.
4 **Wave Detection:** Latest at this stage, the wave description converges to a common level. The selected detection method operates on the trigger times, grouping them into individual wavefronts while being completely agnostic about the type and origin of the original data. The resulting groups of triggers, i.e., waves, are added as another *neo*.Event named ’wavefronts’. Optionally, any number of additional wave descriptions can be calculated and added to the Neo object, including the optical flow vector field or a wave-mode clustering.
5a **Wave-wise Characterization:** The final stage calculates one or multiple characteristic measure(s) of the detected waves. This contains scalar measures as, for example, the wave velocity or its duration, but may also contain metadata information like analysis parameters or information about the dataset added in stage 1 (selected via the 'annotations' block). The output is a pandas dataframe^96^ where each row represents one wave and each column an attribute/characteristic. This pipeline output for one dataset can be directly merged or compared with the output for other datasets and serves as the basis for various cross-domain comparisons (e.g., data comparisons, model validation, method benchmarking).
5b **Channel-wise Characterization:** This alternative final stage is equivalent to the ’Wave-wise Characterization’ in its functionality, but its characteristic measures are calculated per wave and channel (i.e., electrode or pixel). Therefore, in the output dataframe, one row represents one channel for one wave. For either of the two options for the final stage, the characterization can also optionally be performed only on the wave modes instead of on each wave.

##### Data and metadata representation

When designing a pipeline with the objective of modularity and generality, it is of crucial importance to properly define the interfaces between the individual analysis elements (blocks, stages) as well as to the user and other tools. This entails the representation of the data and metadata in a standardized format. For this, we chose the data format Neo.^60^ Neo supports a variety of data types and reading and writing of various common file formats. This interoperability is, thus, ideal for aiding the flexible use of the pipeline. Since Neo itself is very versatile, there are multiple ways how to organize the data and metadata in the Neo structure, so we need to be even more precise in standardizing the data structure. That means that within the pipeline we store the data of all channels in one *neo*.AnalogSignal object and the metadata in the corresponding annotations and array annotations for channel-wise metadata (like their x and y coordinates). Processing and transformation blocks overwrite the data in this Analogsignal object and add corresponding metadata. In stages 3 and 4, additional neo.Event objects may be added to represent transition times and wavefronts as well as an additional AnalogSignal object for derived vector fields (e.g., the optical flow). The file format to use for storing the intermediate results of blocks and stages can be format supported by Neo. We recommend Nix^97^ for a robust file format, or the pickle or numpy for a less robust format that is, however, faster to read and write and produces smaller files.

The entire first stage is dedicated to being the interface between the pipeline and the data resource. It checks whether the data has the required capabilities and then organizes data and metadata into the Neo structure. For the analysis of slow waves with this pipeline, the data needs to be obtained from electrodes or pixels that are arranged on a rectangular grid (which may include empty sites), and that exhibit propagating Up states. The corresponding minimal set of metadata required for the pipeline to process the data are i) the sampling rate, ii) the distance between the electrodes/pixels, iii) and their relative spatial locations of the grid as integer x and y coordinates. Although not explicitly used, it is strongly recommended to include more information such as the measured cortical location, the spatial scale of the grid, the units of the signal, the type and dosage of the anesthetic, an identifier of the dataset, etc. This additional metadata is propagated through the pipeline alongside the data in order to reasonably use and interpret the results.

##### Pipeline interfaces

This degree of flexibility in the execution order of both stages and blocks is based on standardizing the input and output formats. By defining the input requirements for each stage and block, they can successfully interact while remaining interchangeable and thus reusable for other pipelines or applications. Since the individual stages are designed to be potentially reused in other pipelines, the stage outputs, i.e., the intermediate results, should suffice to the same level of completeness and documentation as a final result. Thus, also each stage needs to come with a detailed definition of its input and output structure which is checked by a dedicated ’check_input’ block. These definitions are collected in the stage’s README file to guide developers of alternative pipelines as well as contributors of new blocks for the stage. Similarly, the individual blocks are also thought to serve the modular design by being easy to reuse and recombine, or even used as a standalone application. Therefore, they also need to clearly state the type and format of their in- and outputs. Other than for the stages, this is largely handled organically in form of the dependencies of the corresponding snakemake rule and the definition of the script’s command line arguments and complemented by its docstring.

##### Logging and intermediate results

The modular organization of the pipeline facilitates maintainability, and additional built-in means, such as provenance tracking and storing intermediate results alongside their config files, further support reproducibility, and transparency. Moreover, we emphasize the integration of automatically generated plots of intermediate results. Most blocks produce a plot illustrating their function to make the evolution of the results (or potential bugs) visible. Additional to the config settings, plots, and snakemake logs, we are currently working to further enable the provenance of the analysis results by integrating a formalized provenance tracking with *fairgraph* (https://gin.g-node.org/INM-6/cobrawap_publication_codehttps://pypi.org/project/fairgraph/).

##### Pipeline configuration

The flip side of flexibility and adaptability is complexity and ambiguity. The many combinatorial possibilities need to be controlled by a user interface separate from the actual analysis scripts, e.g., what stages and blocks should be executed, in which order, and with which parameters. Config files (e.g., in csv, yaml, json format) offer human-readable access and control to a user to adapt and execute different variations of the pipeline. Thus, we assign one config file to each stage. Consequently, blocks need to be implemented having generality in mind with any specification handled by corresponding parameters settings, given as command line arguments, i.e., within the pipeline via the config file. Even though this approach is initially more time-consuming, it does pay off in both the quality of the method implementation and its (re-)usability. Furthermore, the availability and aggregation of parameters allow for easier and more transparent calibration of the pipeline across blocks and stages. Additionally, there is a top-level config file for the entire pipeline that specifies the stages and their order and can define global parameters that may also overwrite stage parameters, e.g., for setting the file format or plotting parameters for all stages. Parameters in the config files are typically calibrated for a specific data type or experiment setup. To conveniently switch between calibration presets, the pipeline supports a hierarchical organization of config presets via *profiles*. By executing the pipeline with PROFILE = data1, for each stage the corresponding config file config_data1.yaml is used. For more versatility, profile names can use underscores to define subcategories and exceptions, e.g., data1_subject3. In this case, each stage first looks if a corresponding config file of the same name exists, and if not removes the subcategory with the last underscore from the name, and repeats this lookup until it finds the named config file or defaults to config.yaml. Furthermore, profiles can have variations indicated in the name with a ’|’, e.g., data1_subject3|methodA. This variation key is not removed when first looking up existing config files in the naming hierarchy, only when config|methodA.yaml doesn’t exit it is removed and the lookup loop is repeated.

#### LogMUA estimation *(in stage 2)*

The multi-unit activity (MUA) is an estimate of the local population firing rate, based on the relative spectral power in the high-frequency regime (200–1500 Hz) that can be derived from micro-ECoG recordings.^37^^,^^64^^,^^65^^,^^98^^,^^99^^,^^100^ It is to be noted that not all types of ECoG can detect a MUA signal, depending on the electrode size and impedance, but instead may record an iEEG signal which is not equivalent to MUA. The algorithm for the logMUA estimation first selects a moving window that samples the recording at a given rate. From these samples, the power spectral density (PSD) is calculated using the Welch algorithm. The MUA is defined as the average power in the defined frequency band divided by the average power of the full spectrum. Using the logarithm of the MUA helps to emphasize further the bimodality of the distribution in the presence of slow oscillations. In the selection of the parameters for the algorithm, it is crucial to choose a moving window size large enough so that the chosen frequencies can be accurately estimated ($\text{window}\;\text{size}\geq \frac{1}{\text{highpass}\;\text{frequency}}$) and a corresponding MUA rate so that the full recording is sampled ($\text{MUA}\;\text{rate}\geq \frac{1}{\text{window}\;\text{size}}$). Here, we use a windowsize=0.3s and a MUArate=100Hz. The parameters for the Welch algorithm calculating the PSDs are: samples per segment (nperseg) $=\frac{\text{sampling}\;\text{rate}}{\text{highpass}\;\text{frequency}}$, with overlapping samples (noverlap) =0.5·nperseg (rounded down), using a ’Hann’ window, and a linear detrending.

#### Trigger detection *(in stage 3)*

The pipeline implementation provides multiple options to detect trigger events, i.e., transitions from a low activity state to a high activity state (Up).

• *threshold:* The trigger events can either be defined by setting a threshold value for all the signals or by fitting a bimodal function to the amplitude distribution for each channel in order to set the threshold value. In the latter case, the fitting function is the sum of two Gaussians and the threshold value is set to the central minima. This option is applied to the ECoG datasets in this paper. As an alternative to a double Gaussian fit, there is also the option to only fit the first peak corresponding to the low activity state by only looking at the data left of the peak and defining the threshold as mean+std·SIGMA_FACTOR with a user-defined SIGMA_FACTOR. Since the thresholding method detects also the corresponding downward transitions, this block is usually paired with an additional block that removes Up and Down states that are too short, given user-defined minimal Up and Down durations.
• *Hilbert-phase:* Instead of detecting threshold crossings on the actual signal, the upstrokes of the upward transitions can be detected by thresholding the phase signal of the corresponding analytic signal. An adequate threshold value is a matter of definition, here, we apply −π/2, which corresponds well to the beginning of the upstroke in the actual signal. To be more robust, the algorithm only selects time points where the threshold is crossed from smaller to larger values and where the crossing is followed by a peak (phase =0). This option is applied to the calcium imaging datasets in this paper unless otherwise indicated. For an accurate estimation of the Hilbert-phase it is crucial that the signals are z-scored and detrended to prevent any offset or drift.
• *minima:* As a third option, we adapted and improved the minima detection method presented in.^72^ This method relies on the assumption that in an adequately filtered signal that the existence of a local minimum followed by a peak of a certain height indicates the start of an upward transition. This is particularly suitable for recording techniques characterized by a fast characteristic rise time (i.e., comparable with the theoretical minimum time interval between the passage of two waves on a single channel, e.g., optical data). We improved this method by including some further refinement on trigger candidates. Under the assumption that only one minima candidate can lie between two ”good” local maxima candidates, we impose that 1) local maxima candidates need to have a signal intensity higher than a relative threshold value, determined in a moving window; 2) local maxima candidates need to be separated by a minimum distance (associated with the characteristic frequency of the investigate phenomenon); 3) a local minima candidate needs to be followed by a monotonically rising signal for a defined time interval (also associated to the characteristic frequency of the investigated phenomenon). If more than one candidate minimum is found between two local maxima candidates, the last one before the following ”good” maxima is selected.

#### Trigger clustering *(in stage 4)*

Wavefronts are defined as clusters of trigger times in the three-dimensional space of the electrode arrangement (x,y) and samples in time (t). To run a clustering algorithm in this space, the units of the time dimension need to be translated to the units of the spatial dimensions. The ideal transformation factor (TIME_SPACE_RATIO) depends on the expected dynamics of the phenomena. A wave that propagates linearly with $v_{0}$ is best recognized in the cluster when the time dimension is transformed by a factor $v_{0}/\left(\text{sampling}\;\text{rate}\times \text{spatial}\;\text{scale}\right)$. Thus, if we expect a propagation velocity roughly in the range of $∼10-20\frac{mm}{s}$ then the transformation factor for the calcium imaging data with sampling rate 25 Hz and spatial scale 50μ m is $∼8-16\frac{pixel}{frame}$. Here, we choose a TIME_SPACE_RATIO of 11 for the calcium imaging data which scales according to the spatial resolution to a factor of 0.25 for the logMUA ECoG data with a sampling rate of 100 Hz. The clustering is performed by a density-based algorithm (scipy.cluster.DBSCAN), illustrated in Figure S7. The additional parameters for this algorithm are the minimum number of samples (MIN_SAMPLES_PER_WAVE) and the typical distance between neighboring sample points (NEIGHBOUR_DISTANCE) and were determined by calibrating test recordings from both calcium imaging and ECoG data and scaled consistently with the spatial resolution.

#### Optical flow estimation *(in stage 4)*

The optical flow is the pattern of apparent motion in a visual scene, which here corresponds to the recorded signal on the recording grid. To estimate the optical flow of the spatial propagation of activation, we apply the Horn-Schunck algorithm with a quadratic penalty function and a 3x3 Scharr derivative filter on the phase of the signal (the alternative application using the signal’s amplitude, as well as different derivative filters can be selected via the configuration). Although other penalty functions, i.e., the Charbonnier function, are more accurate, we found that here the simple quadratic function is sufficient. This observation is in agreement with Townsend et al. (2018)^61^ who report good results for the near quadratic edge case of the penalty function. Their study also guided our choice of the parameter α=1.5, determining the weight of the smoothness constraint over the brightness constancy constraint. The resulting vector field is smoothed by a Gaussian kernel which reflects the dimensions of the expected wave activity with respect to the spatial and temporal scale of the data.

### Quantification and statistical analysis

#### Kernel estimation

The kernel estimations for the plotted distributions in Figures 5 and 6 use scipy.stats.gaussian_kde with the default Scott’s rule^101^ as bandwidth method, except for the distributions of inter-wave intervals which use 0.2 times the standard deviation as the kernel size.

#### Velocity filter

Since the channel-wise velocity measure can produce unreasonably high values when there are near identical time delays between spatially distant triggers, we cap the presented distributions at 120 mm/s.

#### Effect size

The effect sizes (*ES*) for the method comparisons in section “Interchangeable blocks enable benchmarking of methods” between two sets of sample values (*A*, *B*) are calculated according to Hedges (1981)^102^ as

$${\text{ES}}_{AB}=\frac{\left|<A>-<B>\right|}{\sigma_{AB}^{\text{pooled}}}$$

$$\sigma_{AB}^{\text{pooled}}=\sqrt{\frac{\left(N_{A}-1\right)\cdot \sigma_{A}^{2}+\left(N_{B}-1\right)\cdot \sigma_{B}^{2}}{N_{A}+N_{B}-2}}$$

using the samples’ average (<>), standard deviation (σ), and number (*N*).

## Supplemental information

Document S1. Figures S1–S7

Video S1. Wave video of example ECoG recording, related to Figure 4

Video S2. Wave video of example calcium imaging recording, related to Figure 4

Document S2. Article plus supplemental information
