# Supplementary material for: A modular and adaptable analysis pipeline to compare slow cerebral rhythms across heterogeneous datasets
Source: Cell Rep Methods. 2024 Jan 5;4(1):100681. doi: 10.1016/j.crmeth.2023.100681 (PMC10831958; doi:10.1016/j.crmeth.2023.100681)
Supplement: Document S1. Figures S1–S7 [file mmc1.pdf]

**Cell Reports Methods, Volume *4***

## Supplemental information

# A modular and adaptable analysis pipeline

# to compare slow cerebral rhythms

# across heterogeneous datasets

**Robin Gutzen, Giulia De Bonis, Chiara De Luca, Elena Pastorelli, Cristiano Capone, Anna Letizia Allegra Mascaro, Francesco Resta, Arnau Manasanch, Francesco Saverio Pavone, Maria V. Sanchez-Vives, Maurizio Mattia, Sonja Grün, Pier Stanislao Paolucci, and Michael Denker**

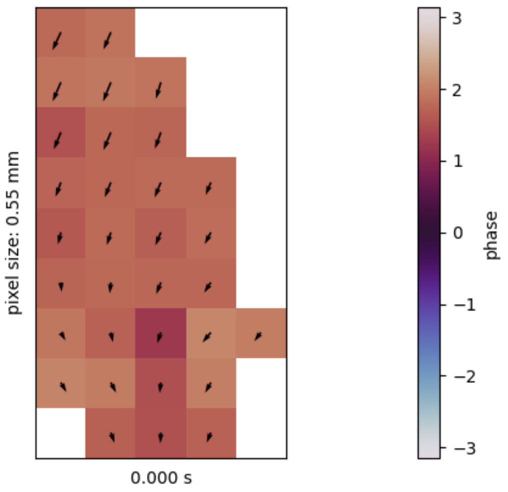


Video S1: **Wave video of example ECoG recording, Related to Figure 4.** The figure shows the first frame of the video showing the wave activity as it is output from stage 4 of the Cobrawap pipeline. The corresponding video is available in the online version of this paper.

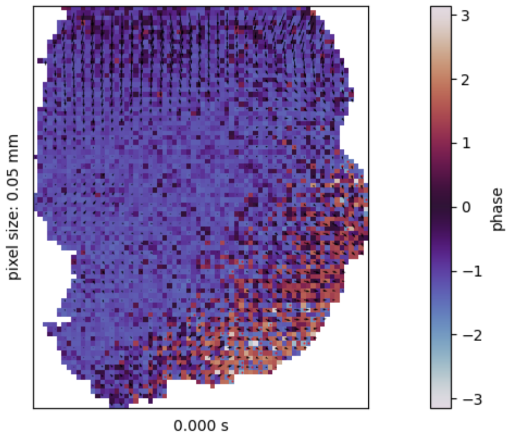


Video S2: **Wave video of example calcium imaging recording, Related to Figure 4.** The figure shows the first frame of the video showing the wave activity as it is output from stage 4 of the Cobrawap pipeline. The corresponding video is available in the online version of this paper.

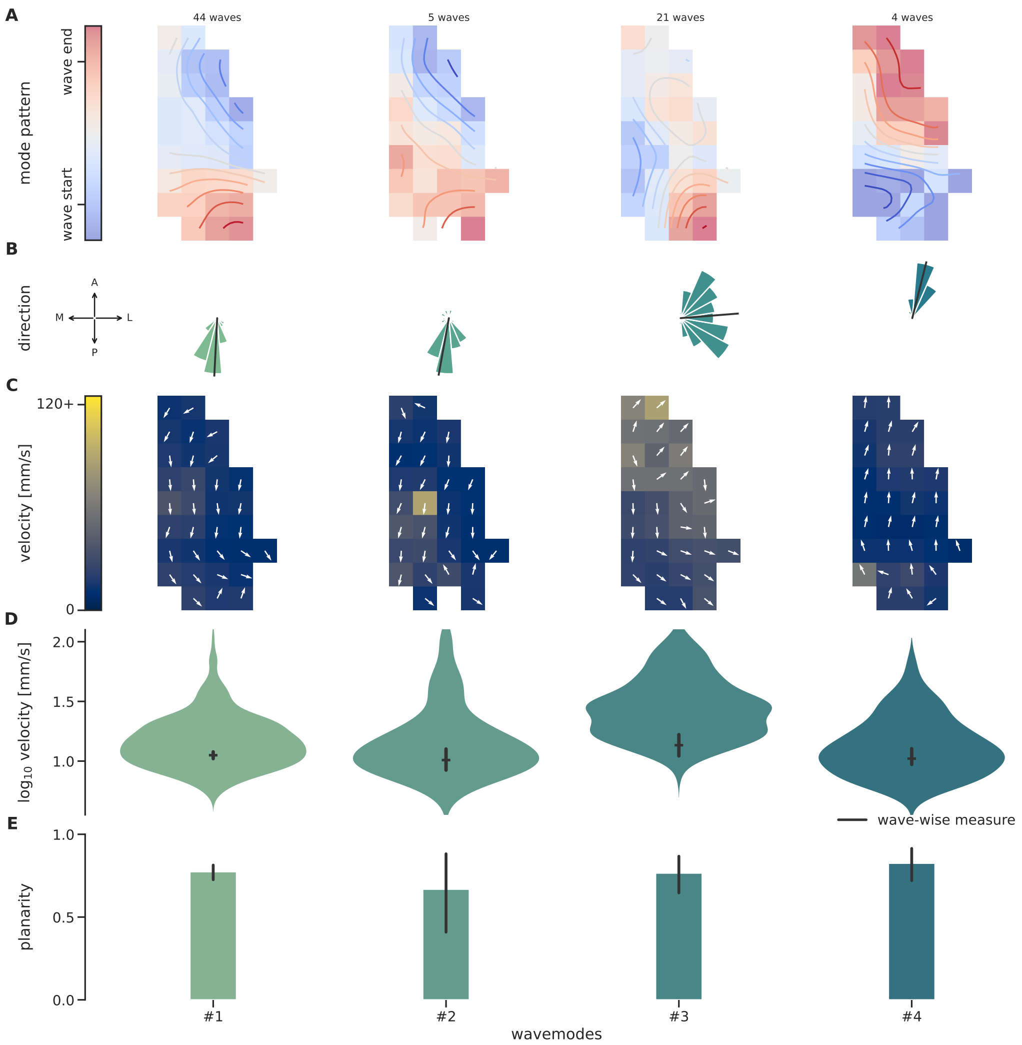


Figure S3: **Characterization of wave-modes in one ECoG recording, Related to Figure 4.** Within the pipeline, the optional block 'wave_mode_clustering' groups together similar wave modes. Their characterization of the waves in each of the 4 modes is shown in the corresponding columns. **A:** The average wave pattern (number of waves indicated on top) is illustrated as a time-delay heatmap with iso-delay contours. **B:** The aggregated histogram of channel-wise directions in waves of this mode. The black lines indicate the average wave-wise direction measure. **C:** Map of the average channel-wise velocities in waves of this mode, overlayed with the average channel-wise direction determined via the optical flow. **D:** The corresponding distributions of channel-wise velocities and as black ticks and errorbars the average and 95% CI of the corresponding wave-wise velocities. **E:** The average and 95% CI of the planarity values for the waves of this mode. The figure is analogous to Figure 4.

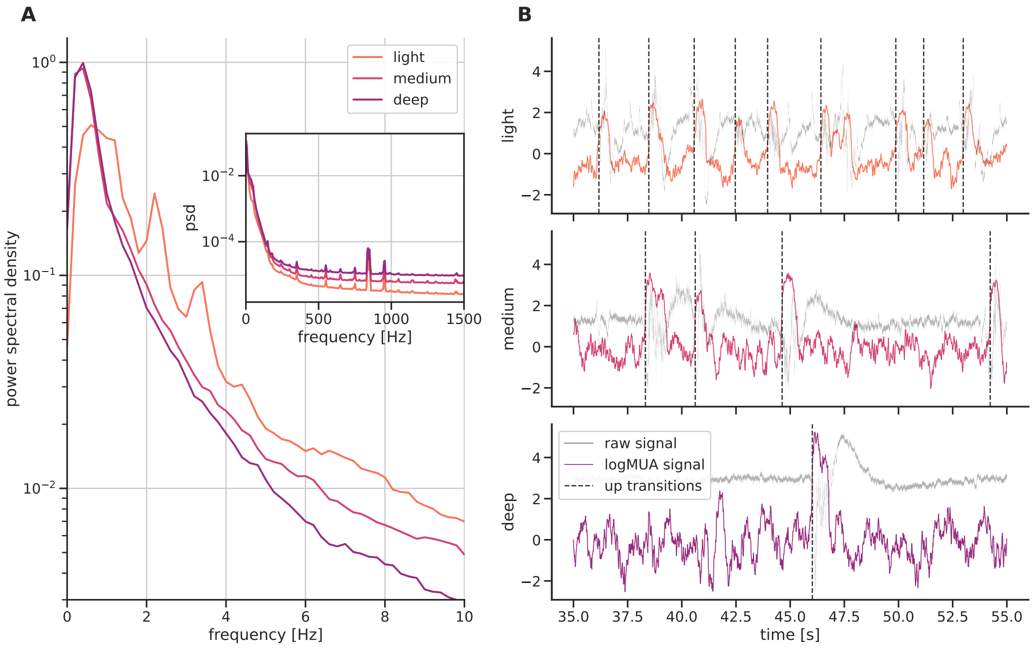


Figure S4: **Detecting up transitions at different anesthesia levels in ECoG recordings, Related to STAR Methods.** Using the same datasets as presented in Figure 5A. **A:** The average power spectral density of the raw ECoG signals shows the changes in spectral power dependent on the anesthesia level. **B:** Three 20 s recording snippets of an example channel during light, medium, and deep anesthesia, respectively. The raw signal (grey) is transformed to a logMUA signal (colored) by using the logarithm of the average spectral power in 200-1500 Hz (see Methods) to detect the transitions to Up states (black dotted).

| measurement technique | anesthetic | anesthetic dosage | disease model | subject | recording length |
|---|---|---|---|---|---|
| ECoG | ketamine & medetonidine | 75 & 13 mg/kg | WBS KO | WBS_KO_6 | 170 s |
| ECoG | ketamine & medetonidine | 75 & 13 mg/kg | WT | WBS_WT_2 | 122 s |
| ECoG | ketamine & medetonidine | 75 & 13 mg/kg | WT | WBS_WT_5 | 140 s |
| ECoG | ketamine & medetonidine | 100 & 13 mg/kg | WBS KO | WBS_KO_1 | 168 s |
| ECoG | ketamine & medetonidine | 100 & 13 mg/kg | WBS KO | WBS_KO_2 | 132 s |
| ECoG | ketamine & medetonidine | 100 & 13 mg/kg | WBS KO | WBS_KO_3 | 129 s |
| ECoG | ketamine & medetonidine | 100 & 13 mg/kg | WBS KO | WBS_KO_4 | 172 s |
| ECoG | ketamine & medetonidine | 100 & 13 mg/kg | WBS KO | WBS_KO_5 | 137 s |
| ECoG | ketamine & medetonidine | 100 & 13 mg/kg | WBS KO | WBS_KO_6 | 170 s |
| ECoG | ketamine & medetonidine | 100 & 13 mg/kg | WT | WBS_WT_1 | 124 s |
| ECoG | ketamine & medetonidine | 100 & 13 mg/kg | WT | WBS_WT_3 | 164 s |
| ECoG | ketamine & medetonidine | 100 & 13 mg/kg | WT | WBS_WT_4 | 138 s |
| ECoG | isoflurane | 80 mg/kg | FXS KO | FXS_KO_1 | 197 s |
| ECoG | isoflurane | 80 mg/kg | FXS KO | FXS_KO_2 | 106 s |
| ECoG | isoflurane | 80 mg/kg | FXS KO | FXS_KO_3 | 243 s |
| ECoG | isoflurane | 80 mg/kg | WT | FXS_WT_3 | 208 s |
| ECoG | isoflurane | 90 mg/kg | WT | FXS_WT_4 | 151 s |
| ECoG | ketamine & medetonidine + iso | 55 & 1.3 mg/kg + 0% | WT | PM_WT_1 | 228 s |
| ECoG | ketamine & medetonidine + iso | 55 & 1.3 mg/kg + 0% | WT | PM_WT_2 | 198 s |
| ECoG | ketamine & medetonidine + iso | 55 & 1.3 mg/kg + 0% | WT | PM_WT_3 | 205 s |
| ECoG | ketamine & medetonidine + iso | 55 & 1.3 mg/kg + 0% | WT | PM_WT_4 | 205 s |
| ECoG | ketamine & medetonidine + iso | 55 & 1.3 mg/kg + 0% | WT | PM_WT_6 | 201 s |
| ECoG | ketamine & medetonidine + iso | 55 & 1.3 mg/kg + 0% | WT | PM_WT_7 | 199 s |
| ECoG | ketamine & medetonidine + iso | 55 & 1.3 mg/kg + 0% | WT | PM_WT_8 | 211 s |
| ECoG | ketamine & medetonidine + iso | 55 & 1.3 mg/kg + 0.2% | WT | PM_WT_2 | 202 s |
| ECoG | ketamine & medetonidine + iso | 55 & 1.3 mg/kg + 0.2% | WT | PM_WT_3 | 220 s |
| ECoG | ketamine & medetonidine + iso | 55 & 1.3 mg/kg + 0.2% | WT | PM_WT_6 | 216 s |
| ECoG | ketamine & medetonidine + iso | 55 & 1.3 mg/kg + 0.5% | WT | PM_WT_1 | 212 s |
| ECoG | ketamine & medetonidine + iso | 55 & 1.3 mg/kg + 0.5% | WT | PM_WT_4 | 204 s |
| ECoG | ketamine & medetonidine + iso | 55 & 1.3 mg/kg + 0.5% | WT | PM_WT_7 | 204 s |
| ECoG | ketamine & medetonidine + iso | 55 & 1.3 mg/kg + 0.5% | WT | PM_WT_8 | 210 s |
| ECoG | ketamine & medetonidine + iso | 55 & 1.3 mg/kg + 0.75% | WT | PM_WT_3 | 213 s |
| ECoG | ketamine & medetonidine + iso | 55 & 1.3 mg/kg + 1.0% | WT | PM_WT_1 | 198 s |
| ECoG | ketamine & medetonidine + iso | 55 & 1.3 mg/kg + 1.0% | WT | PM_WT_2 | 213 s |
| ECoG | ketamine & medetonidine + iso | 55 & 1.3 mg/kg + 1.0% | WT | PM_WT_4 | 210 s |
| ECoG | ketamine & medetonidine + iso | 55 & 1.3 mg/kg + 1.0% | WT | PM_WT_6 | 209 s |
| ECoG | ketamine & medetonidine + iso | 55 & 1.3 mg/kg + 1.25% | WT | PM_WT_7 | 221 s |
| ECoG | ketamine & medetonidine + iso | 55 & 1.3 mg/kg + 1.25% | WT | PM_WT_8 | 211 s |
| calcium imaging | ketamine & xylazine | 100 & 10 mg/kg | WT | M2 | 40 s |
| calcium imaging | ketamine & xylazine | 100 & 10 mg/kg | WT | M2 | 40 s |
| calcium imaging | ketamine & xylazine | 100 & 10 mg/kg | WT | M2 | 40 s |
| calcium imaging | ketamine & xylazine | 100 & 10 mg/kg | WT | M2 | 40 s |
| calcium imaging | ketamine & xylazine | 100 & 10 mg/kg | WT | M2 | 40 s |
| calcium imaging | ketamine & xylazine | 100 & 10 mg/kg | WT | M2 | 40 s |
| calcium imaging | ketamine & xylazine | 100 & 10 mg/kg | WT | M2 | 40 s |
| calcium imaging | ketamine & xylazine | 100 & 10 mg/kg | WT | M2 | 40 s |
| calcium imaging | ketamine & xylazine | 100 & 10 mg/kg | WT | M3 | 40 s |
| calcium imaging | ketamine & xylazine | 100 & 10 mg/kg | WT | M3 | 40 s |
| calcium imaging | ketamine & xylazine | 100 & 10 mg/kg | WT | M3 | 40 s |
| calcium imaging | ketamine & xylazine | 100 & 10 mg/kg | WT | M3 | 40 s |
| calcium imaging | ketamine & xylazine | 100 & 10 mg/kg | WT | M3 | 40 s |
| calcium imaging | ketamine & xylazine | 100 & 10 mg/kg | WT | M3 | 40 s |
| calcium imaging | ketamine & xylazine | 100 & 10 mg/kg | WT | M3 | 40 s |
| calcium imaging | ketamine & xylazine | 100 & 10 mg/kg | WT | M3 | 40 s |
| calcium imaging | isoflurane | 1-2% | WT | 190109 | 318 s |
| calcium imaging | isoflurane | 1-2% | WT | 190109 | 320 s |
| calcium imaging | isoflurane | 1-2% | WT | 190109 | 320 s |
| calcium imaging | isoflurane | 1-2% | WT | 191022 | 300 s |
| calcium imaging | isoflurane | 1-2% | WT | 191022 | 300 s |
| calcium imaging | isoflurane | 1-2% | WT | 191022 | 300 s |

Figure S5: **Data overview, Related to STAR Methods.** Each row shows one of the 60 recordings used in this study. The columns show some of the attributes in which they can differ, and within each column, different values are colored differently.

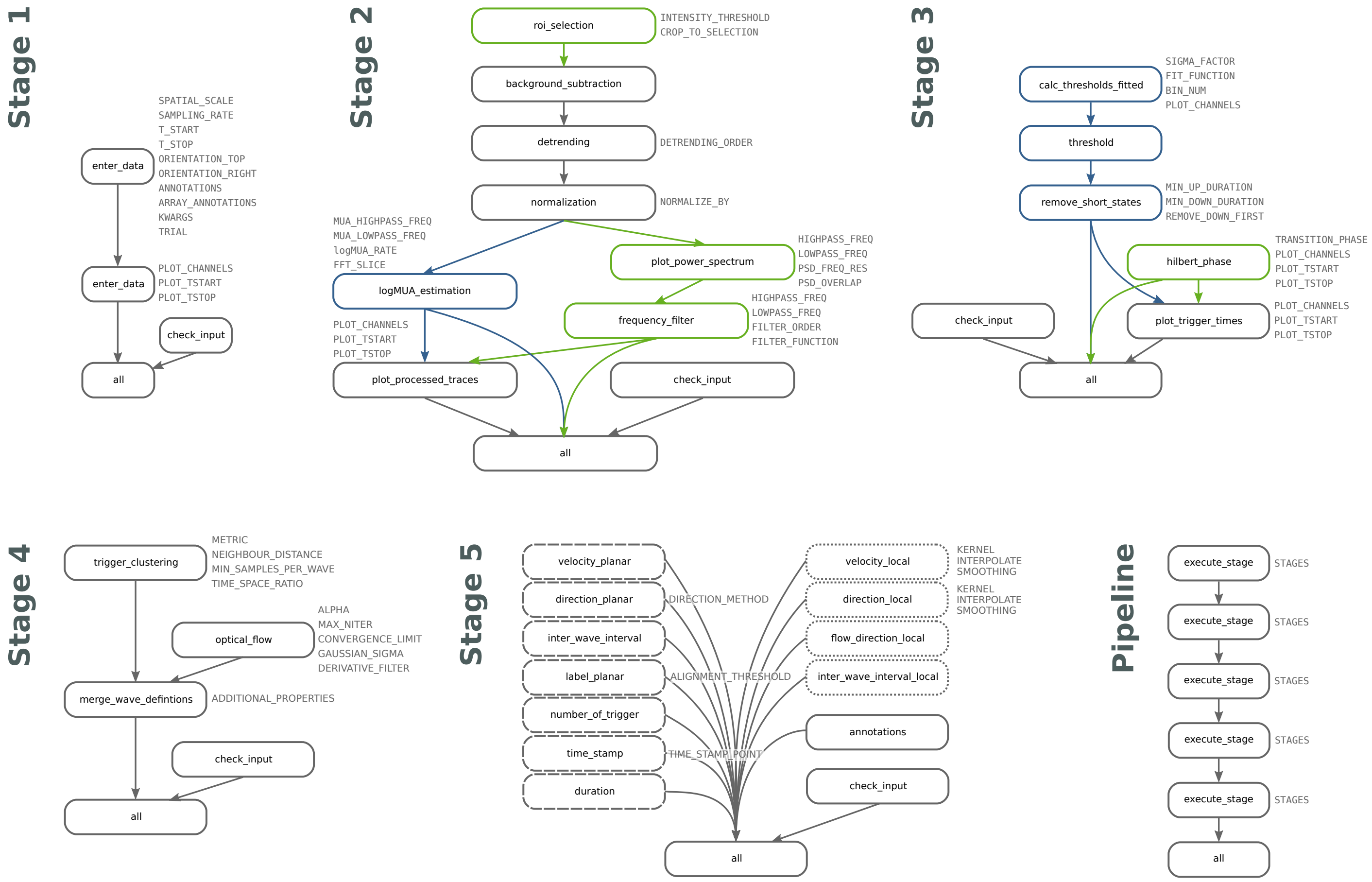


Figure S6: **Pipeline structure, Related to STAR Methods.** The diagrams show the execution order of the blocks for each stage (plus the full pipeline) as generated by the snakemake workflow management framework. Blocks that are specific for the ECoG data are shown in blue, and blocks specific for calcium imaging in green, while common blocks are grey. In stage 5, dashed blocks are wave-wise measures and dotted blocks are channel-wise measures. Next to the blocks, the parameters are indicated that can be set in the corresponding config files.

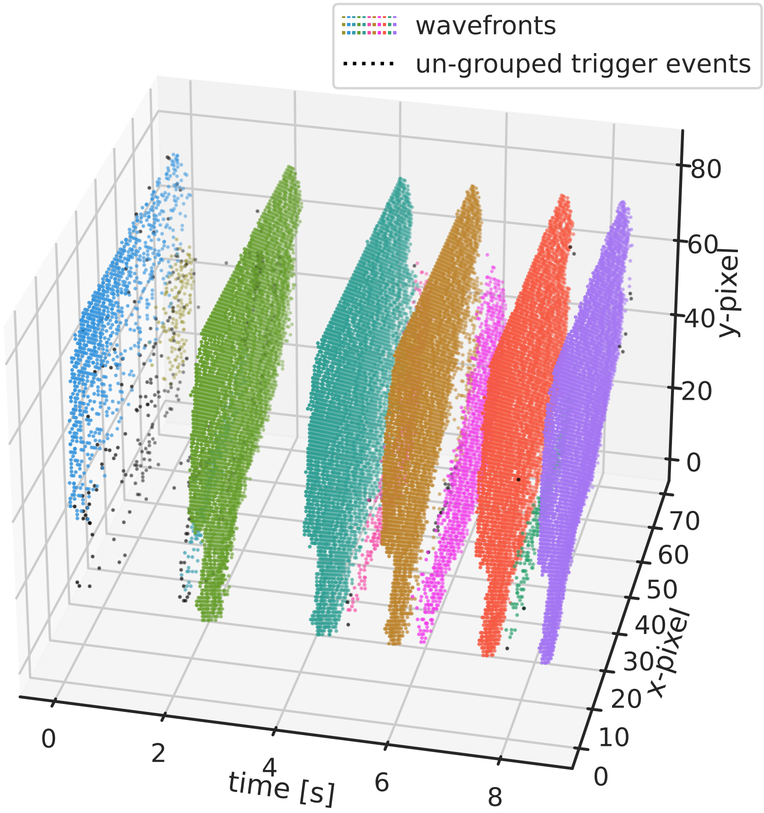


Figure S7: **Wavefront definition via trigger clustering, Related to STAR Methods.** Visualizing the clustering of detected transition times in the space-time domain for 10 s of an example calcium imaging recording. The trigger events are grouped based on their proximity in space and time using a density-based clustering algorithm (color coded).
